# Position paper on postgraduate medical education on the occasion of hospital reform – postgraduate medical education must be considered. A joint position paper by Bündnis Junge Ärztinnen und Ärzte and AG Junge Gastroenterologie of the DGVS as well as the Young DGN

**DOI:** 10.3205/zma001677

**Published:** 2024-04-15

**Authors:** Eckhart G. Hahn

**Affiliations:** 1 University Hospital Erlangen, Department of Medicine 1, Erlangen, Germany

**Keywords:** position paper postgraduate medical education, Bündnis Junge Ärztinnen und Ärzte (BJÄ, Alliance of Young Physicians in Germany), postgraduate medical education on Germany, hospital reform in Germany, hospital transparency law in Germany, German Association for Postgraduate Medical Education

## Abstract

The Bündnis Junger Ärztinnen und Ärzte (BJÄ, Alliance of Young Physicians in Germany) has presented a position paper (PP) on Postgraduate Medical Education (PGME) against the background of an unfolding hospital reform in Germany, and they describe existing deficits of PGME in Germany. Based on this, demands were made of legislators, employers and medical associations which could result in a sweeping reformation of PGME. Hospital reforms can only be accomplished with well trained and motivated physicians. In this respect the BJÄ regards the reform of hospitals and the health-care system as a chance for a reform of PGME, which is long overdue. Legislative competence for PGME lies with the States of the Federal Republic of Germany and this warrants an adjustment of state medical association laws to accommodate the demands of the BJÄ. Generally PGME must be taken into consideration in all health-care legislation, in analogy to the meanwhile globally adopted principle of “Health in all Politics (HiAP)”. The BJÄ has made every endeavour to produce this PP. The responsible stakeholders and actors in the health-care system would be well-advised to take the position paper seriously with a dwindling physician work force in hospitals and serious quality deficits in PGME. Hence, the BJÄ must be comprehensively supported. They need congenial partners to define the scientific foundation of all their demands, to test their application under real life conditions in hospital and outpatient care, to pursue research on the impact on patient care and on the intended transformation of the health-care system. This might best be accomplished by partnering with a scientific *Association for Postgraduate Medical Education* as has been the case in many countries for decades.

## Abstract (de)

Das Bündnis Junger Ärztinnen und Ärzte (BJÄ) hat ein Positionspapier (PP) zur ärztlichen Weiterbildung (ÄWB) vor dem Hintergrund der sich entwickelnden Krankenhausreform vorgelegt, das bestehende Defizite der ÄWB in Deutschland beschreibt. Daraus werden Forderungen an Gesetzgeber, Arbeitgeberinnen und Arbeitgeber sowie Ärztekammern abgeleitet, die eine umfassende Erneuerung der ÄWB bewirken würde. Gesundheitsreformen sind nur mit gut vorbereiteten und motivierten Fachärztinnen und Fachärzten zu bewältigen. Insofern betrachtet das BJÄ die Reformen im Krankenhaus- und Gesundheitswesen als eine Chance für eine längst überfällige Reform der ÄWB. Die Gesetzgebungskompetenz der Bundesländer für die ÄWB macht es erforderlich, die Heilberufe und Kammergesetze der Länder mit Hilfe der Gesundheitsministerkonferenz an die Forderungen des BJÄ anzupassen. Flankiert werden muss dies durch die Berücksichtigung der ÄWB in allen Gesetzen im Gesundheitswesen, ähnlich dem inzwischen global anerkannte Prinzip „Health in all Politics (HiAP)“.

Das BJÄ hat eine große Anstrengung unternommen, indem sie das PP erarbeitet haben. Die verantwortlichen Akteure im Gesundheitswesen wären gut beraten, das PP vor dem Hintergrund von schwindenden ärztlichen Personalressourcen und ernsthaften Qualitätsmängeln in der ÄWB ernst zu nehmen. Das BJÄ muss umfassend unterstützt werden. Die wissenschaftliche Begründung aller geforderten Maßnahmen, die Testung in der realen Umgebung von Krankenhäusern und der ambulanten Krankenversorgung, die wissenschaftliche Begleitung der Auswirkungen auf die Versorgung von Patienten und die angestrebte Transformation des Gesundheitswesens braucht kongeniale Partner. Am wirkungsvollsten könnte dies von einer wissenschaftlichen Gesellschaft für Ärztliche Weiterbildung geleistet werden, wie dies in vielen Ländern seit Jahrzehnten der Fall ist.

## Introduction

The PP of the Bündnis Junge Ärztinnen und Ärzte (BJÄ, Alliance of Young Physicians in Germany) in co-operation with the AG Junge Gastroenterologie der DGVS (Working Group Young Gastroenterology of the German Association for Digestive and Metabolic Diseases) and the Young DGN (German Association for Nuclear Medicine) [1] creates a sensation! 

For the first time young physicians of all specialisations, who are directly affected by laws and statutes of Postgraduate Medical Education (PGME) in Germany have commented on their situation, have comprehensively described the settings and challenges of PGME, analysed it and expressed demands on legislators, employers and professional medical associations. According to their statement the BJÄ [https://www.buendnisjungeaerzte.org], as of November 2023, incorporate 35 scientific and professional medical associations; this gives a strong thrust for their advance. How will the demands of the PP possibly be put into effect?

## Hospital reform and PGME in Germany

The PP was triggered by the forthcoming hospital reform, as it was laid down in a White Paper of July 10, 2023 between the German ministry of health of the Federal Republic of Germany and all German federal states [2]. Based on this, the bill for the advancement of quality in hospital care by transparency (hospital transparency act) [3] is of major significance for the implementation of this process. The Bundesrat (upper house of the German parliament) referred the bill to the arbitration commission in order to have it fundamentally revised [4]. The arbitration commission has dealt with the bill on February 22, 2024 and confirmed it without changes. It has now been accepted by Bundesrat on 22.03.2024. Legislative procedures or plans for a multitude of further bills are pursued [https://www.bundesgesundheitsministerium.de/service/gesetze-und-verordnungen.html]. 

Minister of Health Karl Lauterbach aspires to nothing less than a revolution, a ground-breaking improvement of patient care. But: without a reformation of PGME this cannot be successful! The future generation of physicians will have to implement such radical consequences of new laws and they will have to be prepared for it! Aspects of PGME are not visible in almost all of the new bills for the hospital reform and indeed – as observed in the PP – the impression is that many aspects of PGME were forgotten. 

The evolution of PGME in Germany has a long history, with a fascinating description in the so called “Facharzturteil” (decision for physician specialists) of the German Federal Constitutional Court [5]. It was ruled, that the *legislative competence for PGME lies with the federal states* and not with the federal government. Furthermore, it was also ruled that* boundaries must be set for regulations by professional medical associations* because federal states must remain at the helm of legislation for certain *norms for PGME*. One of these explicitly denominated norms is the *general role of physician specialists within the entire health system*!

Minister Lauterbach had to respect the problem of legislative competence. When federal states would not accept many of his propositions, he developed a system of quality indicators, for which the federal government had legislative competence according to art. 72, section 2 of the German constitution. For example, in the hospital transparency act in art. 1 (amendment of the 5^th ^book of social law books) as well as in art. 2 (amendment of hospital payment law) the number of physicians with their specialist denomination and the number of physicians in PGME including specialty will have to be reported. Up to now there are no such reports of physicians in PGME. Hence, in spite of rulings by the German federal high court of justice in relation to the problem of novice medical procedures [6] it was not possible to survey the safeguarding of specialist standards for patient care by physicians in PGME for each hospital and in Germany as a whole. Now it will be possible to estimate the simultaneity factor (this is the sum of one PGME position and the fraction of a specialist position to cover novices and safeguard the delivery of specialist standards). This has a strong influence on the physician workforce, particularly on the number of specialists in relation to physicians in PGME! The new calculation tool for hospital-based physician workforces of the German medical association (ÄPB-BÄK) [7] this is not yet exemplified. The plan is, to calculate additional 0,2 physician for every resident in the 1^st^ and 2^nd^ year of PGME, and 0,1 physician for 3^rd^ and 4^th^ year residents, and no addition for the 5^th^ and 6^th^ year of PGME (communicated by Prof. Dr. Hendrik Herrmann and Dr. Johannes Gehle, chairmen of the standing conference “postgraduate medical education” of the German medical association and [8]). The application in real life will show, whether this calculation will be applicable to all disciplines and for each type of hospital to secure the safety of patients according to the ruling cited in reference [6].

An insurmountable obstacle for a structured PGME in German hospitals is the lack of dedicated budgets. Almost all demands in the PP of the BJÄ have consequential costs which must be taken from DRG revenues. DRGs include the costs of physicians but not the costs of structured PGME! In this respect the hospital reform and the transformation of the health-care system offer challenges and chances for an adequate financing of PGME, thereby satisfying the needs of structures, processes and outcomes of a competence-based future PGME. This would also meet the demands of the PP of the BJÄ. It would also have to be clarified, whether the German federal government might have legislative competence in this context according to art. 74, section 1, number 12 and art. 72, section 2 of the German constitution. Calculation models for costing were suggested and tested [8], [9], p. 156. Costs for PGME would have to be included in hospital maintenance costs. 

## Outlook

A legal basis is needed to enforce the demands of the BLÄ on legislators, employers, and medical associations. In view of the legislative competence for PGME lying with the German federal states, priority should be given to tackle the health professions laws for a forward-looking PGME. Moreover, PGME must be taken into consideration in all health-care legislation, in analogy to the meanwhile globally adopted principle of “Health in all Politics (HiAP)”! The BJÄ has demonstrated how despite an almost superhuman professional burden an important result was achieved by co-operation. Nonetheless there are good reasons why scientific associations for PGME are active in many countries. In Germany the *commission for PGME of the Gesellschaft für Medizinische Ausbildung* (GMA, DACH Association for Medical Education, [https://gesellschaft-medizinische-ausbildung.org/ausschuesse/weiterbildung.html]) aims at quality improvement of PGME and has published relevant suggestions [10]. The bilingual (German, English) GMS Journal for Medical Education (impact factor 1.6) is available for papers related to PGME. The extraordinary diverse challenges to attain the demands of the BJÄ deserve a broad scientific and organizational support such as could be provided by a German Association for Postgraduate Medical Education. Target groups of such an association are physicians in PGME and all other groups in the health-care system. This would support the generation of physician specialists who are well prepared for the accountability of a changing health-care system in Germany. Everybody should support the demands in the PP of the BJÄ! 

## Competing interests

The author declares that he has no competing interests.
